# Explainable Dual‐Stream Graph Learning With Attention Fusion for Multi‐Stage Hepatitis C Classification

**DOI:** 10.1049/htl2.70107

**Published:** 2026-10-27

**Authors:** Abdul Aziz Patwary, Sharifun Nahar Priya, Md. Ashiqur Rahman, Mohammod Ashikur Rahman, Md. Khairul Islam

**Affiliations:** ^1^ Department of Computer Science and Engineering Bangladesh University of Business and Technology (BUBT) Dhaka Bangladesh; ^2^ Department of Computer Science and Engineering International University of Business Agriculture and Technology (IUBAT) Dhaka Bangladesh

**Keywords:** deep graph neural network, explainable AI, Hepatitis C, SMOTE, sequential backward elimination

## Abstract

Hepatitis C is a chronic liver disease that can progress to fibrosis, cirrhosis and liver failure, requiring accurate multi‐stage classification. This study proposes an explainable dual‐stream deep graph neural network for five‐class classification using 615 patient records from the UCI Hepatitis C dataset. A leakage‐free pipeline employed two‐stage stratified sampling, normalization, training‐only SMOTE and sequential backward elimination, producing 350 training, 81 validation and 184 independent test samples. The framework integrates a clinical branch for biomarker representation with a KNN‐based graph branch using attention‐enhanced residual graph learning, followed by adaptive attention and multi‐scale feature fusion. The proposed model achieved 98.91% accuracy, 98.95% precision, 98.91% recall and 98.90% F1‐score, outperforming XGBoost (95.62%) and GAT (95.65%). Ablation analysis demonstrated progressive improvements from branch integration, attention fusion and multi‐scale fusion. Class‐wise ROC‐AUC ranged from 0.9854 to 1.0000. McNemar's exact test showed a statistically significant difference compared with GAT (p = 0.0312). SHAP analysis identified AST, PROT, CHE and ALB as major biomarkers with significant global and stage‐specific effects. Overall, the framework provides accurate and interpretable Hepatitis C stage classification while jointly leveraging clinical and patient‐relational information.

## Graphical abstract

This study presents an explainable dual‐stream graph learning framework for multi‐stage Hepatitis C classification using clinical biomarker data and patient similarity modelling. The proposed architecture integrates clinical and graph representations through attention‐based feature fusion, achieving 98.91% classification accuracy and outperforming conventional machine learning and baseline graph‐based methods. Furthermore, SHAP‐based explainability provides transparent insights into clinically relevant biomarkers, enhancing the interpretability and trustworthiness of the proposed model.

## 1 Introduction

Hepatitis C virus (HCV) is the causative agent of Hepatitis C, which can present as either an acute or chronic infection, ranging from a mild illness to a potentially life‐threatening condition. Chronic HCV causes severe complications like liver cirrhosis and hepatocellular carcinoma (liver cancer). Historically, the cases of hepatitis were not attributed to known viruses were classified as ‘non‐A, non‐B hepatitis,’ a classification proposed in 1970 which later contributed to identify HCV as a distinct virus [1]. The virus is primarily transmitted through exposure to infected blood. For example, the reuse of contaminated syringes, injection drug use, poor hygiene, the use of items contaminated with blood and other blood‐related exposures. According to the World Health Organization (WHO), at least 50 million people worldwide are living with hepatitis C, with approximately 6000 new infections occurring each day [2]. Although this is a huge burden on the world, many people around the world are unaware of it. However, many people do not receive diagnosis and even if they do, effective treatment is not available to many people as well.

Existing diagnostic approaches for HCV are often expensive, time consuming and not widely available, this makes early detection difficult. Due to these constraints, treatment is frequently delayed, raising the likelihood of serious problems like liver cirrhosis has been shown to induce tumours such as hepatocellular carcinoma [3]. Moreover, conventional HCV is not always discovered in the early stages of the disease through screening tests. If a patient is not treated in time, the condition can worsen, causing additional problems. Accurate diagnostic methods and reliable detection of the disease at its early stages are therefore essential.

Artificial intelligence (AI) refers to computational methods that enable machines to perform tasks that typically require human intelligence, such as learning from data, pattern recognition, prediction and decision‐making. Machine learning (ML), a major branch of AI, enables computational systems to learn patterns and relationships from data without explicitly programming every decision rule. In healthcare, AI, ML and deep learning (DL) have been increasingly applied to disease diagnosis, prognosis, risk prediction, medical image analysis, biomarker identification and clinical decision support.

In the context of HCV, ML algorithms can analyse clinical and biomarker characteristics of infection. Clinical and biochemical parameters such as age, sex, albumin (ALB), alanine aminotransferase (ALT) and aspartate aminotransferase (AST) provide important information about liver function and disease progression [4]. With these indicators, machine learning models can analyse medical datasets to identify meaningful patterns associated with HCV infection and disease progression. Furthermore, feature selection techniques can improve classification by removing redundant or less informative variables, thereby improving model efficiency and potentially enhancing predictive performance.

These AI‐driven approaches can support disease screening and clinical decision‐making by enabling the analysis of complex clinical and biochemical patterns. Therefore, the integration of AI and ML provides a promising computational approach for accurate and timely HCV diagnosis and disease‐stage classification.

Recent studies further demonstrate the broad applicability of AI and machine learning across different healthcare domains. Ghaderzadeh et al. developed an explainable extra trees classifier for early detection of type 2 diabetes using demographic, lifestyle, anthropometric, comorbidity and biochemical variables, demonstrating the potential of interpretable machine learning for clinical risk prediction [5]. In another study, Ghaderzadeh et al. systematically reviewed AI applications in polycystic ovary syndrome and reported the use of machine learning, deep learning and explainable AI for diagnosis, biomarker discovery, risk prediction and clinical decision support, while also highlighting challenges related to interpretability, class imbalance, small sample sizes and limited external validation [6]. Similarly, Porkar et al. proposed a self‐organizing map neural network with block processing for brain tumour region detection in noisy MRI images, demonstrating the potential of neural‐network‐based approaches for medical image analysis and tumour localization [7].

Despite the growing application of AI and machine learning in healthcare, important research gaps remain in multi‐stage Hepatitis C classification. Existing HCV studies have demonstrated the effectiveness of conventional machine learning and graph‐based approaches for disease classification and prediction; however, these approaches generally focus either on patient‐level clinical features or relational information rather than jointly learning both types of representations. In particular, the integration of clinical biomarker representations with KNN‐based patient similarity structures through complementary learning streams remains insufficiently explored for multi‐stage HCV classification. Moreover, adaptive fusion of clinical and graph representations using attention mechanisms and multi‐scale feature integration has received limited attention in this context. The combination of feature‐level learning, patient‐level relational modelling and clinically interpretable prediction within a unified framework therefore remains an important research challenge. These gaps motivate the development of a Dual‐Stream framework that can jointly capture patient‐specific clinical characteristics and inter‐patient relationships while providing transparent and reliable disease‐stage predictions.

To address these limitations, we propose a hybrid dual‐stream deep graph neural network (GNN) framework for multi‐stage Hepatitis C classification using clinical and biochemical data. The framework integrates a clinical branch, which learns discriminative feature‐level representations through fully connected layers, and a graph branch, which constructs a KNN‐based patient similarity graph to capture complex inter‐patient relational dependencies through four attention‐enhanced residual GNN blocks. Sequential backward elimination (SBE) is employed for feature selection. The learned representations from both branches are fused through an attention‐based mechanism followed by multi‐scale feature concatenation for robust disease stage classification. Synthetic minority over‐sampling technique (SMOTE) is applied exclusively to the training data to handle class imbalance and SHAP explainable artificial intelligence (XAI) techniques are incorporated to enhance the transparency and clinical interpretability of model predictions.

### 1.1 Key Contributions of This Work

- Proposed dual‐stream deep GNN framework with adaptive feature fusion: We propose a novel dual‐stream deep GNN framework that jointly learns feature‐level and graph‐structured patient representations through parallel clinical and graph branches. An attention‐guided adaptive fusion mechanism with multi‐scale feature integration effectively combines the complementary embeddings, producing a robust representation for accurate Hepatitis C stage classification.
- Leakage‐free evaluation and enhanced performance: The proposed framework achieves an accuracy of 98.91% on the UCI Hepatitis C dataset across five diagnostic categories. A strict leakage‐free evaluation protocol, including train/validation/test splitting prior to preprocessing and training‐only application of SMOTE and SBE, ensures unbiased performance assessment.
- SHAP‐based clinical explainability: To improve transparency and clinical interpretability, the proposed framework incorporates SHAP to identify the most influential clinical biomarkers, such as ALB, ALT and AST, providing reliable feature attribution for Hepatitis C stage classification.

The remainder of the paper is organized as follows: Section 2 reviews the existing literature related to Hepatitis C diagnosis and graph‐based learning approaches. Section 3 presents the problem formulation, while Section 4 describes the proposed methodology and dual‐stream architecture. Section 5 reports the experimental setup, performance evaluation and ablation analysis. Section 6 provides a comparative study with existing state‐of‐the‐art approaches. Section 7 discusses the key findings of the study, followed by the limitations of the proposed framework discussed in Section 8. Finally, Section 9 concludes the paper and outlines future research directions.

## 2 Literature Review

In recent years, machine learning algorithms have gained popularity in the detection of Hepatitis C virus (HCV) with the help of clinical and biochemical data. Traditional classifiers such as Naïve Bayes, k‐nearest neighbours (KNN), support vector machine (SVM) and decision tree showed encouraging results on the UCI HCV dataset, with the latter showing considerable diagnostic precision [4]. The comparative studies also investigated ensemble and boosting methods, with SVM and XGBoost showing competitive results using moderate‐scale datasets of patients [8]. Predicting HCV, cirrhosis and fibrosis with blood biomarkers and deep learning‐based structures was also studied and showed good classification performance [9]. Random Forest models based on ensembles and hybrid classifiers showed better predictive power in HCV diagnosis [10, 11], whereas hybrid predictive and deep learning models demonstrated improved generalization in validation cohorts [12].

More research focused on disease‐stage prediction and comparative multi‐algorithm systems. CART, artificial neural networks (ANN), convolutional neural networks (CNN), logistic regression and ensemble classifiers were used to predict cirrhosis, detect immunoreactivity and analyse treatment response [13, 14, 15, 16]. Optimization‐based boosting models, including OPTUNA, also reported improved predictive performance [17]. The importance of biomarker optimization in HCV analysis was highlighted through feature‐selection‐driven diagnostic systems and QSAR‐based computational drug prediction methods [18, 19]. The efficacy of hybrid machine learning approaches was further demonstrated through fibrosis assessment and ensemble learning systems [20]. Large‐scale comparative and patient‐specific diagnostic studies have also investigated Bayesian networks, neural networks, random forest and hybrid classifiers across clinical datasets [21, 22, 23, 24, 25].

Recent studies have further extended HCV prediction using more advanced machine learning and ensemble learning strategies. Kumari et al. [26] investigated several statistical learning models using the UCI HCV dataset and reported an accuracy of 97.41% with random forest. Similarly, Tusher et al. [27] proposed a bagging‐based machine learning framework with feature selection and class‐imbalance handling, achieving 98.37% accuracy with bagging k‐NN. These recent studies highlight the continued development of data‐driven HCV prediction and emphasize the importance of feature selection and class‐balancing strategies in improving clinical classification performance.

New paradigms of graph‐based learning have introduced relational dependencies in medical data to enhance classification performance [28].

More recently, advanced graph learning architectures have incorporated attention and Transformer mechanisms to model complex relationships in medical data. Zhang et al. [29] introduced a dynamic graph Transformer framework for medical image classification, demonstrating the potential of dynamically modelling disease relationships within graph structures. Graph Transformer‐based approaches have also been investigated for medical prediction tasks, where Transformer‐based message passing enables the model to capture both local neighbourhood information and longer‐range dependencies [30]. A multi‐task graph Transformer further demonstrated that jointly learning related clinical prediction tasks can improve graph‐based representation learning and predictive performance [30]. These recent developments indicate a growing shift from conventional GNNs towards more expressive attention‐based graph architectures. However, the application of such advanced graph learning strategies to patient‐level multi‐stage Hepatitis C classification remains limited.

XAI‐based systems and real‐time smart monitoring systems further evidenced the possibility of optimizing feature processing, interpretability and transparency in clinical decisions [31].

Furthermore, responding to recent trends in bioinformatics and computational biology, advanced machine learning architectures have been applied to graph‐structured biological data. GCNs and graph embedding techniques, such as DeepWalk and Node2vec combined with neural collaborative filtering, have been used to predict miRNA and lncRNA‐disease associations [32, 33, 34, 35]. These approaches are further complemented by matrix factorization methods [36] and transfer learning utilizing BioBERT embeddings [37]. Such developments demonstrate the broader applicability of graph‐based representation learning to complex biomedical relationships.

The recent progress in graph‐based biomedical learning provides a strong foundation for applying relational representation learning to clinical datasets. In particular, patient similarity graphs can provide a mechanism for representing dependencies among clinically similar individuals, while attention‐based graph architectures can selectively emphasize informative relational patterns. Nevertheless, existing HCV studies have predominantly focused on conventional machine learning, ensemble models or isolated feature‐based prediction, with comparatively limited investigation of advanced graph‐based relational learning for multi‐stage HCV classification.

By reviewing the existing literature, we identified the following research gaps:

- Reliance on conventional machine learning models: The majority of studies still rely on classical machine learning algorithms such as SVM, random forest and decision trees, which may have limited capacity to capture complex relationships in clinical data.
- Insufficient feature optimization: Many existing works use the complete feature set without adequate feature selection, which may introduce redundancy and reduce model efficiency.
- Dataset imbalance and early detection issues: Class imbalance and subtle biomarker variations, particularly in early disease stages, can negatively affect classification performance.
- Limited relational and explainable learning: Although some studies have explored graph‐based models and explainable AI, the joint use of patient‐level relational information, attention‐based feature fusion and quantitative explainability for multi‐stage HCV classification remains limited.

In addition, despite the recent development of advanced graph Transformer and attention‐based graph architectures, their use for multi‐stage HCV classification remains insufficiently explored. Existing approaches also provide limited integration of clinical feature learning, patient‐level relational representation, attention based fusion and quantitative explainability within a unified framework.

To overcome these shortcomings in current HCV detection studies, we propose a hybrid diagnostic framework that combines clinical machine learning and graph‐based representation learning. Standard classifiers are initially used to establish baseline performance, followed by SBE for feature selection. A patient similarity graph is then constructed to represent relational dependencies among patient records, while the proposed dual‐stream architecture integrates clinical and graph‐based representations through attention‐based fusion. The resulting framework is designed to improve classification performance, generalization and interpretability for multi‐stage Hepatitis C classification.

## 3 Problem Formulation

Assume that $D={\left\{\left(x_{i},y_{i}\right)\right\}}_{i=1}^{N}$ is the clinical data that will be used to identify the HCV with $x_{i}$ representing an individual patient and $y_{i}$ represents the disease class label of $x_{i}$. The feature vectors $x_{i}\in R^{12}$ are comprised of biochemical and demographics such as the main liver biomarkers that can be clinically important in the diagnosis of HCV.

The class label space is defined as:

(1) $$\begin{array}{cc} y_{i}\in C= & \;\left\{\text{Blood Donor},\;\text{Suspect Blood Donor},\right.\\ & \left.\text{Hepatitis},\;\text{Fibrosis},\;\text{Cirrhosis}\right\} \end{array}$$

This study aims at learning a discriminative mapping functionality that can correctly categorize samples of patients at various stages of HCV infection:

(2) $$f_{\theta }:R^{12}\to C,$$

Here, θ represents parameters that one can learn. Using the probabilistic approach to learning, the classifier determines the posterior probability distribution:

(3) $$P_{\theta }\left(y|x\right),$$

Lastly, the most probable label is obtained by the use of maximum A posteriori (MAP) decision rule:

(4) $$\hat{y}=\arg\max_{c\in C}P_{\theta }\left(y=c|x\right).$$

In order to maximize the model parameters, the training process minimizes the categorical cross‐entropy loss function that is characterised by:

(5) $$L\left(\theta \right)=-\frac{1}{N}\sum_{i=1}^{N}\sum_{c=1}^{\left|C\right|}1\left(y_{i}=c\right)\;\log P_{\theta }\left(y=c|x_{i}\right).$$

In this formulation, 1(.) is the indicator function and allows the predictive structure to model complicated nonlinear interactions among clinical biomarkers and robustly complete multi‐stage classification of HCV progression.

## 4 Proposed Methodology

### 4.1 Methodology Overview

Figure 1 illustrates the proposed methodology as a structured and leakage‐free framework for multi‐class Hepatitis C disease stage classification. The process begins with dataset splitting into training and testing subsets to ensure unbiased model evaluation. Subsequently, data preprocessing is performed, including normalization and preparation of clinical features. To address class imbalance, SMOTE is applied exclusively to the training data, followed by feature selection using SBE to identify the most informative clinical attributes and reduce feature redundancy. The selected features are then provided as input to the proposed dual‐stream architecture, which simultaneously learns feature‐level representations through a clinical branch and graph‐based relational representations through a graph branch. The learned embeddings from both branches are integrated using attention‐based fusion, multi‐scale feature fusion, and feature aggregation to generate a comprehensive representation for disease stage classification. Finally, XAI techniques are employed to provide transparent, interpretable and clinically meaningful insights into the model predictions.

**FIGURE 1:**
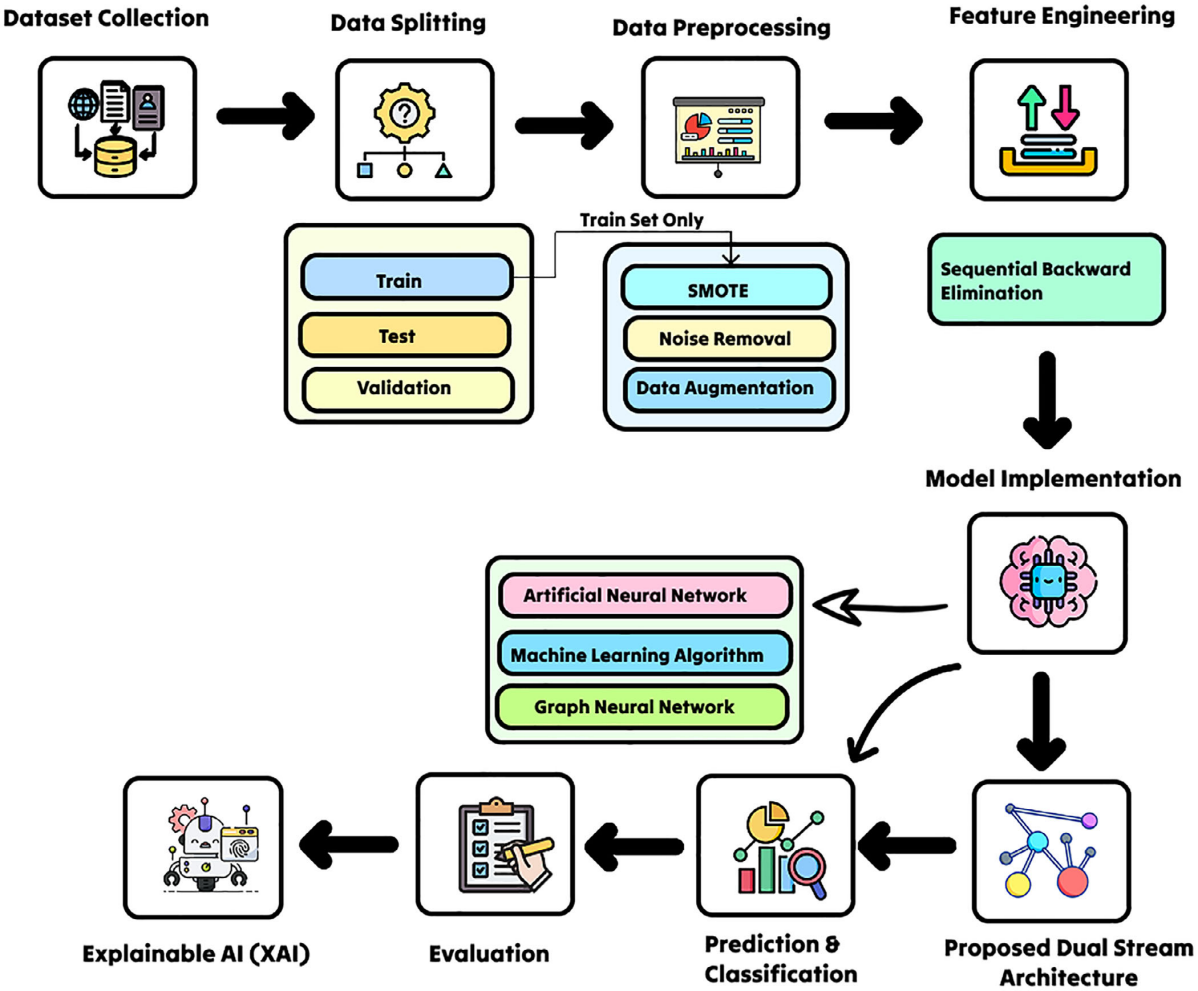
Overview of the proposed methodology for multi‐class Hepatitis C stage classification. The pipeline performs data preprocessing, class balancing, and feature selection, followed by the proposed dual‐stream architecture for classification with SHAP‐based explainability.

### 4.2 Dataset Description

The dataset used in this study is a publicly available blood biomarkers for Hepatitis C Virus Detection dataset obtained from the UCI Machine Learning Repository. It consists of five diagnostic categories: Blood donor, suspect blood donor, Hepatitis C, fibrosis and cirrhosis. The original dataset contained 615 patient records with a highly imbalanced class distribution, where the suspect blood donor and fibrosis classes were significantly under‐represented.

### 4.3 Dataset Splitting Strategy

To ensure fair, reliable and leakage‐free evaluation, the dataset was partitioned using a two‐stage stratified sampling strategy. The 615 patient records were first divided into a development set (70%, n=431) and an independent test set (30%, n=184). The development set was then further partitioned into training (n=350) and validation (n=81) subsets. This yields a final stratified partition of 350 training, 81 validation and 184 test samples (56.9%/13.2%/29.9%). Stratification was applied at both stages to preserve the class distribution across the subsets.

The independent test set was kept completely unseen during model training, feature selection and hyperparameter tuning. SMOTE and SBE were applied exclusively to the training data, while the validation set was used for model selection and hyperparameter tuning. The independent test set was reserved exclusively for final performance evaluation.

### 4.4 Pre‐Processing

Let the original Hepatitis C dataset be denoted as

(6) $$D={\left(X_{i},y_{i}\right)}_{i=1}^{N},$$

where $X_{i}\in R^{F}$ represents the clinical feature vector of the i‐th patient and $y_{i}\in 1,2,3,4,5$ denotes the diagnostic class label corresponding to blood donor, suspect blood donor, Hepatitis C, fibrosis and cirrhosis.

Each patient record consists of biochemical and demographic attributes. The complete feature matrix can be expressed as

(7) $$X\in R^{N\times F},$$

where N denotes the number of patients and F represents the total number of selected features.

To ensure a leakage‐free evaluation protocol, the dataset is first partitioned into training, validation, and testing subsets as

(8) $$D=D_{\text{train}}\cup D_{\text{val}}\cup D_{\text{test}}$$

where

(9) $$D_{\text{train}}\cap D_{\text{val}}=\emptyset ,$$

(10) $$D_{\text{train}}\cap D_{\text{test}}=\emptyset ,$$

(11) $$D_{\text{val}}\cap D_{\text{test}}=\emptyset .$$

Feature normalization is subsequently applied to the training, validation, and testing subsets to ensure numerical consistency:

(12) $$X_{\text{norm}}=\frac{X-\mu }{\sigma },$$

where μ and σ denote the mean and standard deviation of each feature, respectively.

The normalized training dataset is represented as,

(13) $$D_{\text{train}}^{\text{norm}}=\left\{\left(X_{i}^{\text{norm}},y_{i}\right)\right\}$$

the normalized validation dataset is represented as

(14) $$D_{\text{val}}^{\text{norm}}=\left\{\left(X_{i}^{\text{norm}},y_{i}\right)\right\}$$

and the normalized testing dataset is represented as

(15) $$D_{\text{test}}^{\text{norm}}=\left\{\left(X_{i}^{\text{norm}},y_{i}\right)\right\}$$

To address severe class imbalance, the SMOTE function S(·) is applied exclusively to the normalized training dataset. For a minority sample $x_{i}$, synthetic samples are generated as

(16) $$x_{\text{new}}=x_{i}+\lambda \left(x_{nn}-x_{i}\right)$$

where $x_{nn}$ represents a selected nearest neighbour of $x_{i}$ belonging to the same class and λ∼U(0,1) is a random interpolation factor.

The oversampling objective is defined as

(17) $$\Delta =\max_{c}|D_{c}|-\min_{c}\left|D_{c}\right|$$

where $|D_{c}|$ denotes the number of samples belonging to class c.

The resampling process continues until balanced class representation is achieved within the training dataset:

(18) $$|D_{c}^{\text{train}}|=N_{b},\;\forall c\in \left\{1,2,3,4,5\right\}$$

where $N_{b}$ denotes the final balanced sample size per class.

The balanced training dataset is therefore expressed as

(19) $$D_{\text{train}}^{\text{bal}}=S\left(D_{\text{train}}^{\text{norm}}\right)$$

The validation and testing datasets remain completely unchanged and are never subjected to oversampling or feature‐selection procedures. The validation set is used for model selection and hyperparameter tuning, whereas the testing set is reserved exclusively for final performance evaluation on unseen samples.

This preprocessing pipeline ensures normalized feature representation, balanced training data distribution, robust model validation and leakage‐free evaluation, providing a reliable foundation for feature engineering and the proposed dual‐stream architecture for Hepatitis C classification.

### 4.5 Feature Selection Using Sequential Backward Elimination

SBE was employed to reduce feature redundancy and retain the most informative clinical and biochemical attributes. SBE was applied exclusively to the training data after the initial stratified train/validation/test split, with cross‐validation performed within the training set to prevent information leakage. Starting from the original 12 features, namely age, sex, albumin (ALB), alkaline phosphatase (ALP), alanine aminotransferase (ALT), aspartate aminotransferase (AST), bilirubin (BIL), cholinesterase (CHE), cholesterol (CHOL), creatinine (CREA), gamma‐glutamyl transferase (GGT) and total protein (PROT), SBE iteratively removed less informative features and evaluated the resulting feature subsets. The final subset consisted of 10 features: sex, albumin (ALB), alkaline phosphatase (ALP), alanine aminotransferase (ALT), aspartate aminotransferase (AST), cholinesterase (CHE), cholesterol (CHOL), creatinine (CREA), gamma‐glutamyl transferase (GGT), and total protein (PROT), while age and bilirubin (BIL) were excluded. The selected features were subsequently used as inputs to both the clinical and graph branches of the proposed dual‐stream architecture. The validation and testing sets were kept completely independent of the feature selection procedure and were used only for model selection and final performance evaluation, respectively.

### 4.6 Proposed Dual‐Stream Architecture for Hepatitis C Classification

Figure 2 illustrates the overall architecture of the proposed dual‐stream framework, which integrates a clinical branch and a graph branch for comprehensive disease representation learning. The clinical branch processes selected clinical biomarkers through fully connected layers to extract discriminative feature embeddings, while the graph branch constructs a patient similarity graph and employs residual graph neural network blocks with attention mechanisms to capture complex inter‐patient relationships. The representations learned from both branches are subsequently combined through an attention‐based fusion layer and a multi‐scale fusion module to generate a unified feature representation. Finally, a softmax classifier performs multi‐stage Hepatitis C diagnosis, while SHAP provides interpretable explanations of the model predictions. By jointly learning feature‐level and graph‐structured information, the proposed architecture enhances classification performance and offers a robust and explainable framework for Hepatitis C disease staging.

**FIGURE 2:**
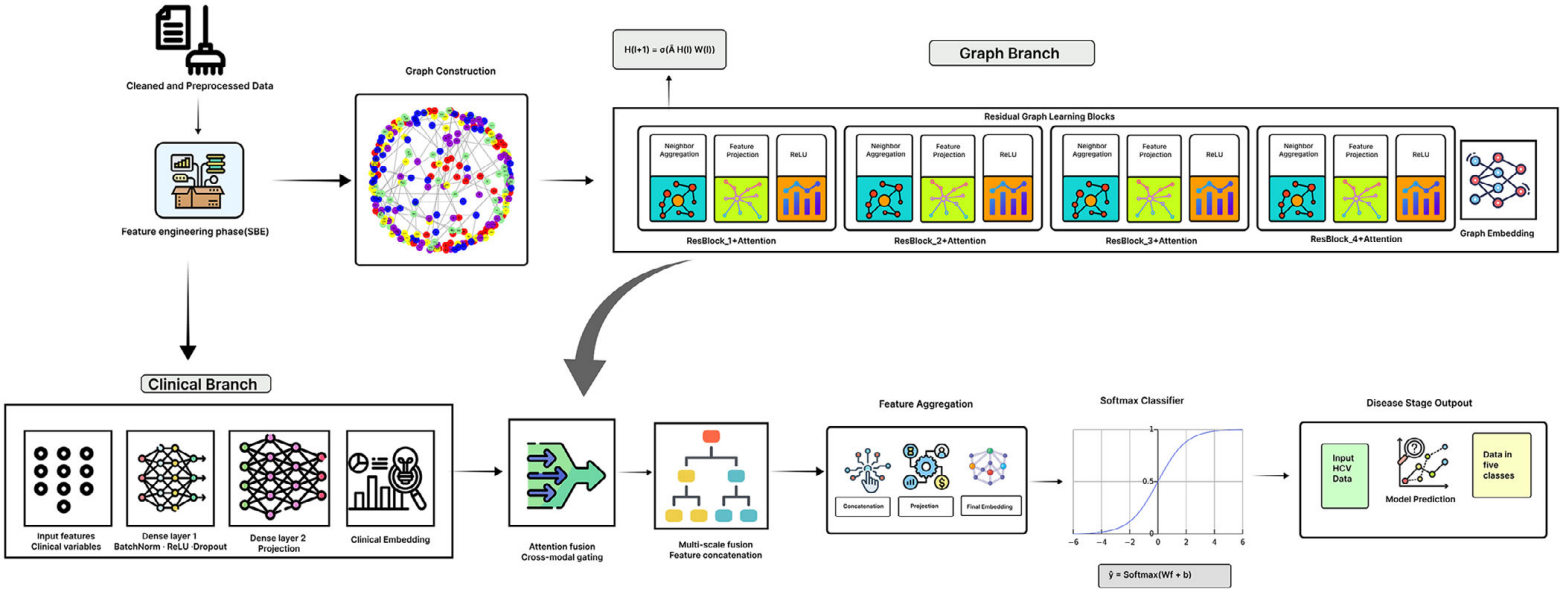
Architecture of the proposed dual‐stream framework for multi‐class Hepatitis C classification. The clinical and graph branches jointly learn complementary feature and relational representations, which are fused for accurate classification and SHAP‐based clinical interpretation.

#### 4.6.1 Overview and Notation

Let the clinical dataset consist of patient samples

(20) $$X={\left\{x_{i}\right\}}_{i=1}^{N},\;x_{i}\in R^{F},$$

where N denotes the total number of patient records and F represents the number of clinical attributes, including biochemical markers and demographic features relevant to Hepatitis C detection.

Each patient sample is associated with a disease class label

(21) $$y_{i}\in \left\{1,2,\text{…},K\right\},$$

where K=5 denotes the diagnostic categories: blood donor, suspect blood donor, hepatitis, fibrosis and cirrhosis.

The proposed framework utilizes a dual‐stream architecture consisting of a clinical branch and a graph branch. The clinical branch learns feature‐level representations directly from the selected clinical biomarkers, while the graph branch captures relational dependencies among patients through a similarity graph.

The objective is to learn a discriminative mapping

(22) $$F_{\theta }:R^{F}\times G\to R^{K},$$

where G=(V,E) denotes the patient similarity graph constructed from clinical features, with V representing the set of patient nodes and E representing similarity‐based connections between patients.

The clinical branch generates clinical feature embeddings, whereas the graph branch employs residual graph neural network layers with attention mechanisms to learn graph‐aware representations. The embeddings from both branches are subsequently integrated through an attention‐based fusion strategy to produce a unified representation for disease stage classification. Finally, a softmax classifier generates class probability predictions, and SHAP is employed to provide interpretable explanations of the model decisions.

#### 4.6.2 Clinical Branch

The clinical branch processes the selected clinical biomarkers through successive dense layers to learn compact and discriminative feature representations.

Given the input feature matrix X, the first dense transformation is computed as

(23) $$H_{c}^{\left(1\right)}=\text{ReLU}\left(\text{BN}\left(XW_{c}^{\left(1\right)}+b_{c}^{\left(1\right)}\right)\right),$$

where $W_{c}^{\left(1\right)}\in R^{F\times 128}$ denotes the trainable weight matrix.

To improve generalization and reduce overfitting, dropout regularization is applied:

(24) $$H_{c}^{(1)*}=\text{Dropout}\left(H_{c}^{\left(1\right)}\right).$$

The second dense layer performs feature projection to obtain the final clinical embedding:

(25) $$Z_{c}=H_{c}^{(1)*}W_{c}^{\left(2\right)}+b_{c}^{\left(2\right)},$$

where

(26) $$Z_{c}\in R^{64}.$$

The resulting clinical embedding captures important biochemical and demographic characteristics associated with different stages of Hepatitis C progression and serves as the feature‐level representation for the subsequent fusion module.

#### 4.6.3 Graph Branch

The graph branch is responsible for learning graph‐aware patient representations from the previously constructed patient similarity graph. Using the graph topology obtained through the KNN‐based graph construction process, the graph branch models both local neighbourhood interactions and higher‐order structural dependencies among clinically similar patients. Unlike the clinical branch, which focuses on individual patient biomarkers, the graph branch explicitly exploits inter‐patient relationships to generate robust relational representations for disease classification.

Given the adjacency matrix A, the normalized graph structure is computed as

(27) $$\tilde{A}=D^{-1/2}\left(A+I\right)D^{-1/2},$$

where D denotes the degree matrix and I introduces self‐loops to preserve node‐specific information during graph message propagation.

The initial node representation is obtained directly from the clinical feature matrix

(28) $$H_{g}^{\left(0\right)}=X.$$

The first graph convolution operation is formulated as

(29) $$H_{g}^{\left(1\right)}=\mathrm{ReLU}\left(\tilde{A}H_{g}^{\left(0\right)}W_{g}^{\left(1\right)}\right),$$

where

(30) $$W_{g}^{\left(1\right)}\in R^{F\times 128}.$$

Subsequent graph feature propagation follows the standard graph convolution operation

(31) $$H_{g}^{\left(l+1\right)}=\sigma \left(\tilde{A}H_{g}^{\left(l\right)}W_{g}^{\left(l\right)}\right),$$

where $H_{g}^{\left(l\right)}$ denotes the node representation at the l‐th graph layer, $W_{g}^{\left(l\right)}$ represents the learnable weight matrix, and σ(·) denotes the nonlinear activation function.

Through iterative neighbourhood aggregation, the graph branch enables each patient node to incorporate information from its neighbouring patients, thereby capturing both direct and higher‐order relational dependencies that cannot be learned from isolated clinical features alone.

The resulting graph representations are subsequently forwarded to the residual graph learning block, where attention‐guided residual propagation further refines the learned structural features before fusion with the clinical branch.

##### 4.6.3.1 Graph Construction and Dual‐Stream Architecture

Given the clinical feature matrix

(32) $$X\in R^{N\times F},$$

where N denotes the total number of patient samples and F represents the number of selected clinical attributes, the proposed framework constructs a patient similarity graph while simultaneously learning feature‐level representations through a dual‐stream learning paradigm.

The patient similarity graph is represented as

(33) G=(V,E),

where V denotes the set of patient nodes and E represents similarity‐based connections among patients.

Graph connectivity is established using the K‐nearest neighbour (KNN) algorithm:

(34) $$A_{ij}=\left\{\begin{array}{cc} 1, & \text{if}\;j\in \text{KNN}(i),\\ 0, & \text{otherwise}. \end{array}\right.$$

To ensure a leakage‐free learning protocol, the patient similarity graph is constructed exclusively from the training subset using the selected clinical biomarkers. Each patient is represented as a node, while edges are established according to the k=5 nearest‐neighbour similarity criterion. The resulting graph serves as the structural input to the graph branch, enabling the proposed framework to capture both local and higher‐order relational dependencies among patients.

Figure 3 illustrates the constructed patient similarity graph used by the proposed dual‐stream architecture. The graph exhibits a globally connected structure with locally clustered regions corresponding to patients having similar clinical characteristics. Such a topology enables the graph branch to effectively propagate information across neighbouring patients while preserving meaningful disease‐specific relationships.

**FIGURE 3:**
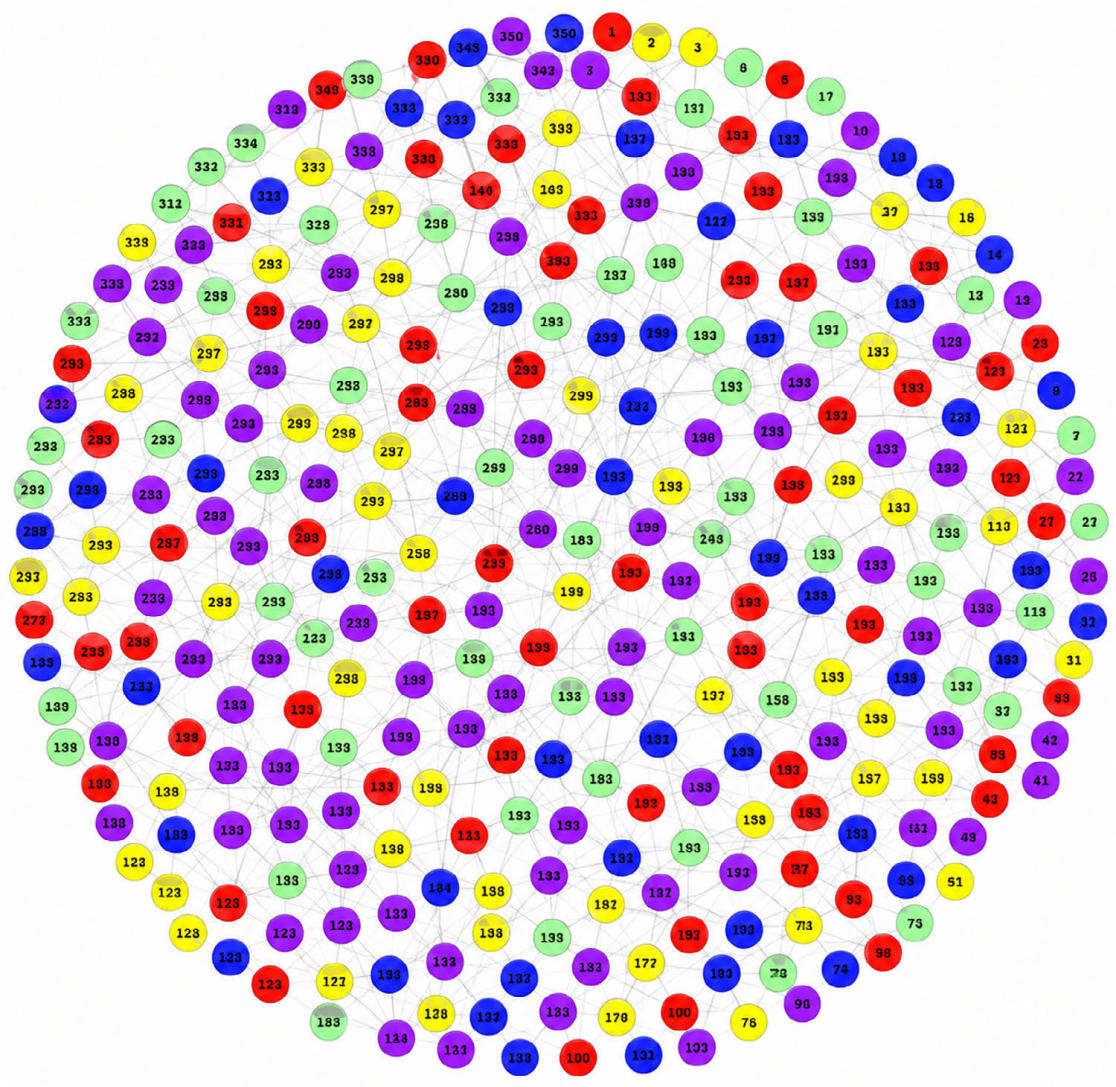
Visualization of the KNN‐based patient similarity graph constructed from the training dataset. Nodes represent patients, edges indicate k=5 nearest‐neighbour relationships, and node colours denote the five Hepatitis C diagnostic categories used by the graph branch for relational learning.

The proposed architecture consists of two complementary learning pathways: a clinical branch and a graph branch. The clinical branch extracts discriminative feature‐level information directly from clinical biomarkers, whereas the graph branch captures higher‐order relational dependencies through graph neural network propagation.

##### 4.6.3.2 Residual Graph Learning Block

To facilitate deeper graph representation learning and alleviate over‐smoothing, four attention‐enhanced residual GNN blocks are employed in the graph branch. Each residual block performs neighbourhood aggregation while preserving previously learned node representations through residual skip connections.

The first residual graph block is formulated as

(35) $$R^{\left(1\right)}=\text{ReLU}\left(\tilde{A}H_{g}^{\left(1\right)}W_{g}^{\left(2\right)}\right),$$

followed by residual propagation:

(36) $$H_{g}^{\left(2\right)}=R^{\left(1\right)}+H_{g}^{\left(1\right)}.$$

The subsequent residual graph blocks follow the generic propagation rule

(37) $$H_{g}^{\left(l+1\right)}={\text{ResBlock}}_{l}\left(H_{g}^{\left(l\right)}\right),\;l=2,\text{…},4,$$

where each residual block consists of graph convolution, attention‐guided feature refinement, and residual feature propagation.

To emphasize diagnostically important structural patterns, an attention mechanism is integrated within each residual block. The attention score is computed as

(38) $$e_{i}=a^{T}\tanh\left(W_{a}h_{i}\right),$$

where $W_{a}$ and a denote trainable attention parameters.

The normalized attention coefficient is given by

(39) $$\alpha_{i}=\frac{\exp(e_{i})}{\sum_{j}\exp\left(e_{j}\right)}.$$

The attention‐weighted graph representation is then obtained as

(40) $$Z_{a}=\sum_{i}\alpha_{i}h_{i}.$$

Finally, the graph embedding is projected as

(41) $$Z_{g}=\text{ReLU}\left(Z_{a}W_{g}^{\left(3\right)}\right),$$

where

(42) $$Z_{g}\in R^{64}.$$

The combination of residual learning and attention‐guided propagation enables effective multi‐hop neighbourhood aggregation while preserving informative node characteristics. Consequently, the graph branch captures both local and higher‐order patient relationships, producing a robust graph representation for subsequent fusion and classification layers.

#### 4.6.4 Attention Fusion Layer

The clinical branch and graph branch learn complementary representations from clinical biomarkers and patient similarity relationships. To effectively integrate these heterogeneous representations, a cross‐modal attention fusion mechanism is employed.

Let

(43) $$Z_{c}\in R^{64}$$

denote the clinical embedding and

(44) $$Z_{g}\in R^{64}$$

denote the graph embedding.

First, an adaptive fusion gate is computed as

(45) $$\beta =\sigma \left(W_{f}[Z_{c}\left|\right|Z_{g}]+b_{f}\right),$$

where $\left[Z_{c}||Z_{g}\right]$ denotes the concatenation of clinical and graph embeddings, $W_{f}$ and $b_{f}$ are trainable parameters, and σ(·) represents the sigmoid activation function.

The attention‐fused representation is then obtained as

(46) $$Z_{f}=\beta Z_{c}+\left(1-\beta \right)Z_{g},$$

where β∈[0,1] dynamically controls the relative contribution of the clinical and graph representations.

This cross‐modal gating mechanism enables the model to adaptively emphasize the most informative features from both branches, resulting in a more discriminative and robust representation for downstream disease stage classification.

#### 4.6.5 Multi‐Scale Feature Fusion

To further enrich representation learning, multi‐scale feature fusion is employed to aggregate information from different representation spaces.

The clinical embedding, graph embedding, and attention‐fused representation are concatenated as:

(47) $$Z_{m}=\text{Concat}\left(Z_{c},Z_{g},Z_{f}\right).$$

This multi‐scale representation preserves complementary information learned by the individual branches while enhancing feature diversity and discriminative capability.

The resulting representation serves as a comprehensive feature descriptor for downstream classification.

#### 4.6.6 Feature Aggregation

Following multi‐scale fusion, the resulting representation $Z_{m}$, as defined in Equation (47), is aggregated to generate a compact and discriminative feature embedding for downstream classification. Here, $Z_{c}$, $Z_{g}$ and $Z_{f}$ denote the clinical embedding, graph embedding and attention‐fused representation, respectively.

The concatenated feature vector is subsequently projected into a lower‐dimensional latent space:

(48) $$Z_{\text{agg}}=\text{ReLU}\left(W_{a}Z_{m}+b_{a}\right),$$

where $W_{a}$ and $b_{a}$ denote the trainable projection parameters.

This projection operation refines the fused representation and produces the final embedding that captures both clinical feature information and graph‐based relational characteristics.

The resulting embedding is then forwarded to the classification head for disease stage prediction.

#### 4.6.7 Classification Head

The aggregated feature representation is processed through a fully connected classification module.

First, a hidden representation is generated as

(49) $$H_{\text{cls}}=\text{ReLU}\left(Z_{\text{agg}}W_{1}+b_{1}\right),$$

where $W_{1}$ and $b_{1}$ denote trainable parameters.

The final class probability distribution is obtained using the softmax function:

(50) $$\hat{y}=\text{Softmax}\left(H_{cls}W_{2}+b_{2}\right).$$

The output layer produces probabilities corresponding to the five Hepatitis C diagnostic categories:

(51) K=5.

The predicted disease stage is determined by selecting the class with the highest posterior probability.

#### 4.6.8 Training Objective

The parameters of the proposed dual‐stream architecture are optimized using the categorical cross‐entropy loss function to minimize the discrepancy between predicted and ground‐truth class labels.

The loss function is defined as

(52) $$L_{\text{CE}}=-\frac{1}{B}\sum_{i=1}^{B}\sum_{k=1}^{K}y_{ik}\log\left({\hat{y}}_{ik}\right),$$

where B denotes the batch size, K represents the total number of diagnostic classes, $y_{ik}$ is the ground‐truth label indicator and ${\hat{y}}_{ik}$ denotes the predicted class probability.

The model parameters are optimized using the Adam optimizer:

(53) θ←Adam(θ,lr,wd),

where lr denotes the learning rate and wd represents weight decay regularization. Minimizing the objective function enables effective learning of both clinical and graph‐based representations for accurate disease stage classification.

#### 4.6.9 Inference Mechanism

During inference, the trained dual‐stream architecture utilizes both clinical biomarkers and graph‐structured patient relationships to generate disease stage predictions.

The overall inference function is expressed as

(54) $$\hat{Y}=F_{\theta }\left(X,G\right),$$

where $F_{\theta }$ denotes the optimized dual‐stream model, X represents the clinical feature matrix and G denotes the patient similarity graph.

The resulting output corresponds to the probability distribution over the five Hepatitis C diagnostic categories.

#### 4.6.10 SHAP‐Based Explainability

To enhance transparency and clinical interpretability, SHAP (SHapley Additive exPlanations) is incorporated to quantify the marginal contribution of each clinical biomarker to the final disease stage prediction.

For a given clinical feature $f_{j}$, the corresponding Shapley value $\phi_{j}$ is computed as:

(55) $$\phi_{j}=\sum_{S\subseteq F\setminus \{j\}}\frac{|S|!(|F|-|S|-1)!}{|F|!}\left[v(S\cup \{j\})-v(S)\right],$$

where F denotes the complete set of clinical features, and v(·) represents the expected model prediction output conditioned on a specific feature subset S.

SHAP provides highly interpretable explanations by evaluating the exact impact of each input variable, identifying the most diagnostically influential biomarkers that drive the final classification decision.

This explainability framework ensures transparent, trustworthy and clinically meaningful decision‐making, which is crucial for reliable application in real‐world diagnostic settings.

#### 4.6.11 Final Prediction

The final disease stage prediction is obtained by selecting the class with the highest posterior probability:

(56) $$\hat{y}=\arg\max_{k}{\hat{Y}}_{k}.$$

The predicted output belongs to one of the five Hepatitis C diagnostic categories, namely blood donor, suspect blood donor, hepatitis, fibrosis or cirrhosis.

By jointly leveraging clinical biomarkers and graph‐structured patient relationships, the proposed dual‐stream architecture effectively captures both feature‐level and relational information, resulting in robust, accurate and interpretable multi‐stage Hepatitis C classification.

### 4.7 Algorithm Overview

Algorithm 1 summarizes the main processing steps of the proposed dual‐stream architecture, from feature preparation and graph construction to classification and explainability. Detailed architectural components and mathematical formulations are provided in Section 4.3.

ALGORITHM 1 **Proposed dual‐stream architecture for Hepatitis C stage classification.**

**Table:** 

1:	**Input**: Normalized clinical feature matrix X and class labels Y.
2:	**Feature selection**: Apply SBE to the training data to obtain the selected clinical features.
3:	**Graph construction**: Construct the patient similarity graph G=(V,E) using KNN with k=5.
4:	**Graph normalization**: Normalize the adjacency matrix and incorporate self‐loops.
5:	**Clinical representation**: Process the selected clinical features through the Clinical Branch to obtain the clinical embedding $Z_{c}$.
6:	**Graph representation**: Process the patient similarity graph through four attention‐enhanced residual GNN blocks to obtain the graph embedding $Z_{g}$.
7:	**Attention‐based fusion**: Adaptively combine $Z_{c}$ and $Z_{g}$ using the attention‐based fusion mechanism to obtain $Z_{f}$.
8:	**Multi‐scale fusion**: Concatenate the clinical, graph, and fused representations to obtain $Z_{m}$.
9:	**Feature aggregation**: Project the multi‐scale representation into a compact aggregated embedding $Z_{agg}$.
10:	**Classification**: Generate the probability distribution over the five diagnostic classes using the classification head.
11:	**Model optimization**: Optimize the model using CrossEntropyLoss and the Adam optimizer.
12:	**Early stopping**: Monitor validation loss and stop training when it does not improve for 10 consecutive epochs, retaining the checkpoint with the lowest validation loss.
13:	**Inference**: Apply the trained model to the unseen test data to obtain class probabilities.
14:	**Explainability**: Apply SHAP to quantify the contribution of the clinical features to the model predictions.
15:	**Output**: Return the predicted Hepatitis C disease stage.

Algorithm 1 provides a concise summary of the complete processing pipeline, while the detailed operations of the Clinical Branch, Graph Branch, residual GNN blocks, attention fusion, multi‐scale fusion, and classification components are described in Section 4.3.

## 5 Experimental Result Analysis

### 5.1 HyperParameter Settings

Table 1 summarizes the optimal hyperparameter configuration adopted for the proposed dual‐stream architecture. The framework simultaneously learns feature‐level representations through the clinical branch and graph‐based relational representations through the graph branch. A systematic hyperparameter tuning strategy was employed to identify the most effective model configuration. The final model utilized four residual GNN blocks with 128 hidden units, while both the clinical and graph embeddings were projected into a 64‐dimensional latent feature space.

**TABLE 1:** Hyperparameter tuning and configuration for the proposed dual‐stream architecture.

Parameter	Candidates tested	Selected value
**Clinical branch**		
Clinical branch hidden units	64, 128	128
Clinical embedding dimension	32, 64, 128	64
Clinical branch activation	ReLU, LeakyReLU	ReLU
Dropout rate	0.3, 0.4, 0.5	0.4
**Graph branch**		
Residual GNN blocks	2, 3, 4	4
Graph hidden units	64, 128	128
Graph embedding dimension	32, 64, 128	64
Graph activation function	ReLU, LeakyReLU	ReLU
Dropout rate	0.3, 0.4, 0.5	0.4
**Feature fusion**		
Fusion mechanism	Attention‐based	Attention‐based
Fusion layer activation	ReLU, LeakyReLU	ReLU
Multi‐scale feature integration	Yes/No	Yes
**Optimization**		
Optimizer	Adam, AdamW	Adam
Learning rate	$1\times 10^{-3}$, $5\times 10^{-4}$	$5\times 10^{-4}$
Weight decay	0.0005, 0.001	0.0005
Batch size	16, 32	32
Maximum epochs	150, 200	200
Early stopping criterion	Validation loss	Patience = 10 epochs
**Graph construction**		
KNN neighbours (k)	3, 5, 7	5
Self‐loop	Yes/No	Yes
Normalization strategy	Symmetric	Symmetric
**Fixed parameters**		
Loss function	CrossEntropyLoss	CrossEntropyLoss
Framework	PyTorch Geometric	PyTorch Geometric
Device	CUDA	CUDA

The clinical branch employed 128 hidden units in its fully connected layers, and ReLU was used as the activation function. The fusion layers also employed ReLU activation to transform the combined representations.

The selected configuration employed a batch size of 32, the Adam optimizer with a learning rate of $5\times 10^{-4}$, and a weight decay of 0.0005. To enhance generalization capability and reduce overfitting, a dropout rate of 0.4 was incorporated during model training.

For graph construction, a 5‐nearest neighbour (KNN) similarity strategy with self‐loop integration was employed to model patient‐to‐patient relationships. The graph branch consisted of four attention‐enhanced residual GNN blocks, enabling effective aggregation of both local and higher‐order neighborhood information. In parallel, the clinical branch learned discriminative feature representations directly from the selected clinical biomarkers through fully connected layers. The learned embeddings from both branches were subsequently integrated using an attention‐based fusion mechanism, followed by multi‐scale feature fusion and feature aggregation to generate a comprehensive disease representation.

The validation set was used for hyperparameter tuning, model selection and training monitoring throughout the optimization process. All candidate configurations reported in Table 1 were evaluated on the validation subset, while the final model performance was assessed exclusively on the unseen test set.

Early stopping was applied by monitoring the validation loss with a patience of 10 epochs, and the model checkpoint corresponding to the lowest validation loss was retained for final evaluation.

This evaluation protocol ensured unbiased model selection and prevented information leakage during the development of the proposed framework.

The selected hyperparameter configuration provided stable convergence and strong generalization performance throughout the training process. Using these optimized settings, the proposed dual‐stream architecture achieved an overall classification accuracy of 98.91% on the Hepatitis C dataset. This result demonstrates the effectiveness of jointly leveraging clinical biomarker information and graph‐structured patient relationships for accurate, robust and interpretable multi‐class Hepatitis C stage classification.

### 5.2 Baseline and State‐of‐the‐Art Comparison

To comprehensively evaluate the effectiveness of the proposed dual‐stream architecture, we compared its performance against both conventional machine learning models and state‐of‐the‐art graph neural network (GNN) architectures. The baseline models include logistic regression, decision tree, K‐nearest neighbours (KNN), support vector machine (SVM), random forest, and XGBoost, which are widely used for clinical data classification tasks. In addition, several representative graph learning architectures, including graph convolutional network (GCN), GraphSAGE, graph attention network (GAT) and graph isomorphism network (GIN), were selected as state‐of‐the‐art graph‐based baselines due to their strong capability in modelling relational dependencies among patients.

To ensure a fair and unbiased comparison, all models were trained and evaluated using the same experimental pipeline, including identical preprocessing procedures, feature selection strategy, SMOTE‐based class balancing, graph construction mechanism, hyperparameter optimization protocol and the same stratified 350/81/184 train–validation–test partition described in Section 4.2. All classification metrics, including precision, recall and F1score, are computed using weighted averaging to appropriately account for the highly imbalanced class distribution present in the Hepatitis C dataset, where the blood donor class substantially dominates over minority classes such as suspect blood donor and fibrosis.

Table 2 presents the comparative results. Among the traditional machine learning methods, logistic regression achieved the lowest performance with an accuracy of 88.43%, while decision tree and KNN improved classification capability by capturing nonlinear relationships within the clinical biomarkers. SVM and random forest demonstrated competitive performance, achieving accuracies of 93.40% and 94.18%, respectively. XGBoost emerged as the strongest traditional baseline with an accuracy of 95.62%, highlighting its effectiveness in learning complex feature interactions from tabular clinical data.

**TABLE 2:** Baseline and state‐of‐the‐art model comparison for Hepatitis C classification.

Model	Acc.	Prec.	Rec.	F1
**Traditional machine learning models**				
Logistic regression	0.8843	0.8890	0.8843	0.8830
Decision tree	0.9084	0.9110	0.9084	0.9080
KNN	0.9175	0.9210	0.9175	0.9170
SVM	0.9340	0.9360	0.9340	0.9335
Random forest	0.9418	0.9450	0.9418	0.9410
XGBoost	0.9562	0.9580	0.9562	0.9560
**Graph neural network models**				
GCN	0.9362	0.9378	0.9362	0.9362
GraphSAGE	0.9445	0.9455	0.9445	0.9442
GIN	0.9512	0.9520	0.9512	0.9510
GAT	0.9565	0.9570	0.9565	0.9562
**Proposed dual‐stream architecture**	**0.9891**	**0.9895**	**0.9891**	**0.9890**

Among the graph‐based models, GCN achieved an accuracy of 93.62% by leveraging patient similarity relationships through graph convolution operations. GraphSAGE further improved performance through neighbourhood aggregation, reaching an accuracy of 94.45%. GIN demonstrated stronger graph representation learning capability with an accuracy of 95.12%, while GAT achieved the best performance among all graph‐based baselines with an accuracy of 95.65%, benefiting from attention‐based message passing and adaptive neighbourhood weighting.

Despite the strong performance of both conventional machine learning and advanced graph neural network models, the proposed dual‐stream architecture consistently outperformed all competing approaches across every evaluation metric. The superior performance can be attributed to the synergistic integration of two complementary learning pathways. The clinical branch learns discriminative patient‐specific biomarker representations, whereas the graph branch captures inter‐patient relational dependencies through attention‐enhanced graph representation learning. Furthermore, the attention‐guided fusion mechanism and multi‐scale feature aggregation strategy enable the framework to effectively combine local clinical information with global graph structural knowledge.

As a result, the proposed dual‐stream architecture achieved the highest classification performance, obtaining an accuracy of 98.91%, precision of 98.95%, recall of 98.91% and F1‐score of 98.90%. These findings demonstrate that jointly leveraging clinical biomarker information and graph‐structured patient relationships provides a more comprehensive representation of Hepatitis C progression patterns than either traditional machine learning models or existing graph neural network architectures. The consistent improvements across all evaluation metrics further confirm the robustness, generalization capability and discriminative power of the proposed framework for accurate and interpretable Hepatitis C stage classification.

### 5.3 Ablation Study

To evaluate the contribution of individual components within the proposed dual‐stream architecture, an ablation study was conducted by progressively incorporating each module into the framework. As shown in Table 3, both the clinical branch and graph branch contributed substantially to classification performance. The integration of the two branches resulted in improved representation learning, while the attention fusion mechanism further enhanced discriminative capability. Moreover, multi‐scale feature fusion provided additional performance gains by effectively combining complementary feature representations. Overall, the complete dual‐stream architecture achieved the highest and most balanced performance, demonstrating the effectiveness of jointly leveraging clinical biomarkers and graph‐structured patient relationships for accurate Hepatitis C stage classification.

**TABLE 3:** Ablation study of the proposed dual‐stream architecture on Hepatitis C classification.

Model variant	Acc.	Prec.	Rec.	F1
Clinical branch only	0.9578	0.9584	0.9578	0.9575
Graph branch only	0.9685	0.9691	0.9685	0.9682
Clinical + Graph branch	0.9794	0.9800	0.9794	0.9791
+ Attention fusion	0.9857	0.9862	0.9857	0.9855
+ Multi‐scale feature fusion	0.9876	0.9880	0.9876	0.9874
**Proposed dual‐stream** **architecture**	**0.9891**	**0.9895**	**0.9891**	**0.9890**

### 5.4 Sensitivity Analysis of KNN Neighbours

To investigate the effect of the neighbourhood size on the performance of the proposed dual‐stream architecture, a sensitivity analysis was conducted by varying the number of nearest neighbours (k) used to construct the KNN‐based patient similarity graph. The values k=3, k=5 and k=7 were evaluated while keeping the remaining model architecture, preprocessing procedure, training protocol and evaluation settings unchanged. The results are summarized in Table 4.

**TABLE 4:** Sensitivity analysis of the KNN neighbourhood size.

KNN (k)	Accuracy (%)	Weighted F1 (%)
3	98.37	98.25
5	98.91	98.90
7	98.11	98.24

As shown in Table 4, the proposed framework achieved its highest performance when k=5 was used, obtaining an accuracy of 98.91% and a weighted F1 score of 98.90%. Reducing the neighbourhood size to k=3 resulted in a decrease in accuracy and weighted F1, with values of 98.37% and 98.25%, respectively. Similarly, increasing the neighbourhood size to k=7 resulted in a further reduction in accuracy to 98.11%, while the weighted F1 score was 98.24%. These results indicate that k=5 provides a suitable balance between capturing local patient relationships and avoiding excessive connections to less similar patients. Therefore, k=5 was retained as the final neighbourhood size for constructing the patient similarity graph.

### 5.5 ROC Curve Analysis

Figure 4 presents the multi‐class receiver operating characteristic (ROC) curves of the proposed dual‐stream architecture for the five Hepatitis C diagnostic categories. ROC analysis was performed to evaluate the discriminative ability of the proposed framework across different classification thresholds. The ROC curves demonstrate high true‐positive rates with relatively low false‐positive rates across the evaluated disease categories, indicating strong class‐wise discrimination.

**FIGURE 4:**
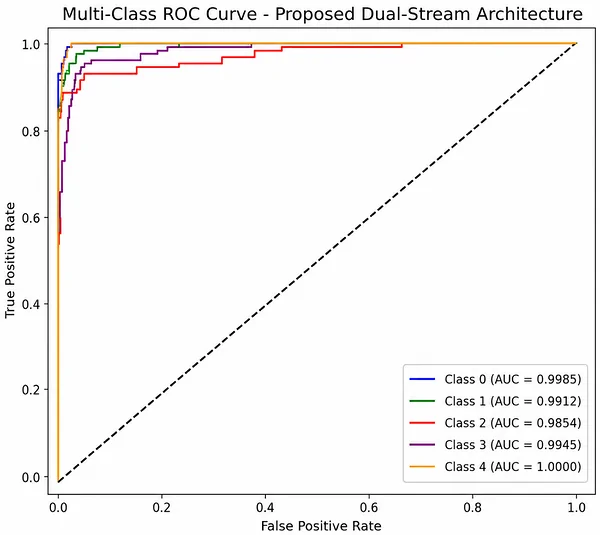
Multi‐class ROC curves of the proposed dual‐stream architecture for Hepatitis C stage classification. The curves illustrate class‐wise discriminative performance across five diagnostic categories, with AUC values ranging from 0.9854 to 1.0000, indicating excellent class‐wise discrimination across the evaluated thresholds.

The proposed framework achieved area under the curve (AUC) values ranging from 0.9854 to 1.0000 across all five diagnostic categories.

The cirrhosis category (Class 4) achieved an AUC of 1.0000, indicating excellent discrimination across the evaluated decision thresholds. However, this result should not be interpreted as evidence of universally perfect classification, particularly because the independent test set contains only nine cirrhosis samples.

The framework also achieved very high AUC values for Class 0 (0.9985), Class 1 (0.9912), Class 2 (0.9854) and Class 3 (0.9945). These results indicate consistently strong discriminative performance across all five diagnostic categories.

The proposed dual‐stream architecture demonstrates strong ROC‐AUC performance compared with the evaluated conventional machine learning and graph‐based baseline models. The consistently high AUC values indicate that the framework can effectively distinguish between the different Hepatitis C disease stages across a broad range of classification thresholds. Unlike evaluation at a single decision threshold, ROC analysis provides a comprehensive assessment of the sensitivity‐specificity trade‐off across varying thresholds.

The high AUC values observed across all five classes further indicate that the learned clinical and graph‐based representations provide strong discriminatory information for disease‐stage classification. Although the cirrhosis class achieved an AUC of 1.0000, although no cirrhosis samples were misclassified on this test set, the class comprises only nine test samples, so an AUC of 1.0000 should be interpreted as evidence of strong discrimination on a small subsample rather than as evidence of perfect separability in general.

Overall, the ROC analysis supports the strong discriminative capability of the proposed framework across the five Hepatitis C diagnostic categories.

### 5.6 Confusion Matrix Analysis

The confusion matrix for the proposed dual‐stream architecture for the five Hepatitis C diagnostic categories: Blood donor, suspect blood donor, hepatitis, fibrosis and cirrhosis is shown in Figure 5. The confusion matrix shows good diagonal dominance, consistent with the majority of patient samples being correctly identified in their respective disease categories. This observation is aligned with the results of high classification accuracy that the proposed framework has obtained.

**FIGURE 5:**
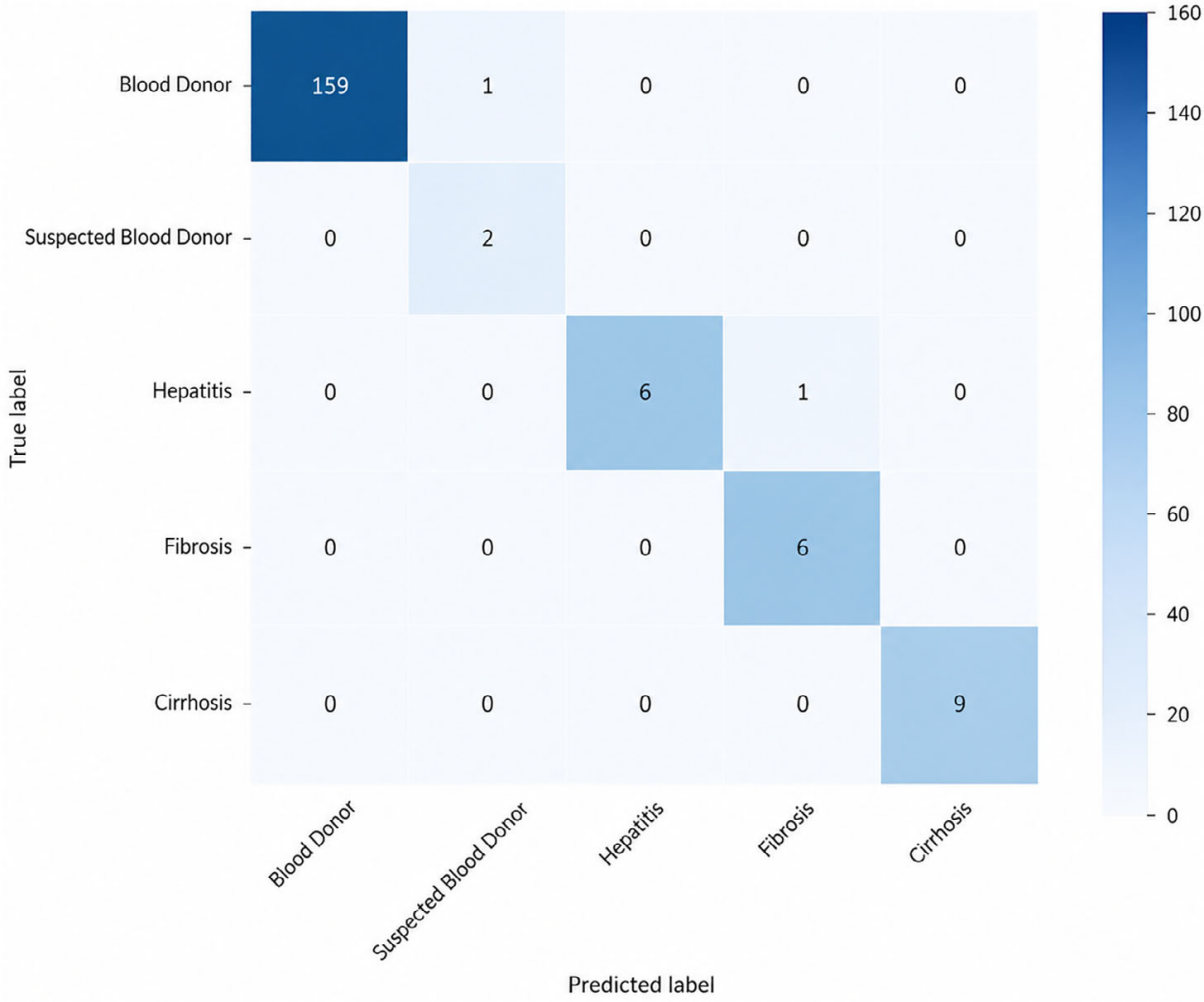
Confusion matrix of the proposed dual‐stream architecture for multi‐class Hepatitis C classification. The matrix exhibits strong diagonal dominance across all five diagnostic categories, indicating high true positive rates and minimal inter‐class misclassification.

A few misclassifications were noted, mostly of the class hepatitis and fibrosis. This is a clinically comprehensible error, since there are biochemical overlaps and indistinguishable clinical pictures during the intermediate stages of the disease. However, these incidences of falsely predicted output were still quite small, indicating that the framework has a good discriminative power.

Interestingly, the model proposed obtained nearly perfect classification for the class cirrhosis, as all nine cirrhosis samples in the test set were correctly classified, although this represents a small subsample. This finding indicated that there is more distinct clinical pattern for the advanced liver disease which can differentiate it from the other disease categories. Overall, the confusion matrix shows that the dual‐stream architecture is successful in effectively integrating the clinical feature representations with the graph‐based patient relationships, yielding powerful and precise Hepatitis C classification for all disease stages.

The limited number of off‐diagonal elements demonstrates the effectiveness of the clinical branch, graph branch and attention‐based fusion mechanism in learning complementary representations for disease classification. Overall, the confusion matrix confirms the robustness, reliability and high classification performance of the proposed dual‐stream architecture for multi‐class Hepatitis C stage prediction.

### 5.7 Statistical Significance Analysis

To further validate the reliability of the observed performance improvement, we conducted McNemar's test which is presented in Figure 6 to compare the proposed dual‐stream architecture with the strongest graph‐based baseline, the GAT. McNemar's test is a widely accepted non‐parametric statistical procedure for determining whether two classifiers exhibit significantly different error rates when evaluated on the same testing samples.

**FIGURE 6:**
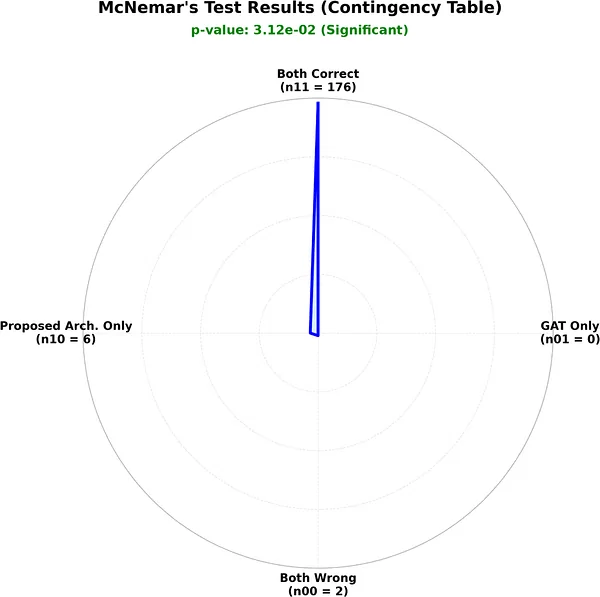
McNemar's test contingency radar chart comparing the proposed dual‐stream architecture and the GAT baseline. The discordant predictions ($n_{10}=6$, $n_{01}=0$) indicate fewer errors for the proposed model on the evaluated test set.

The comparison was performed on the independent testing set. It is important to note that the independent testing subset comprises N = 184 samples (30% of the cohort). Although this is a comparatively generous holdout fraction for a dataset of this size, the absolute number of samples remains small in statistical terms, which constrains the precision of the comparison reported below. Given this constrained evaluation size and the inherently high baseline accuracy of GAT (95.65%), the total pool of misclassified samples is naturally very small. The resulting contingency matrix showed that both models correctly classified 176 instances ($n_{11}=176$) and jointly misclassified only two exceptionally complex instances ($n_{00}=2$). The proposed framework successfully classified the remaining six instances that GAT missed ($n_{10}=6$). Consequently, no instances were exclusively correctly classified by GAT ($n_{01}=0$).

The proposed dual‐stream architecture achieved an accuracy of 98.91%, compared with 95.65% for GAT, corresponding to an observed improvement of 3.26 percentage points. Based on the exact conditional binomial formulation of McNemar's test, the 95% confidence interval for the paired performance difference was [0.27%, 3.26%]. Given the relatively small testing set (N=184) and the limited number of discordant predictions, this observed improvement should nevertheless be interpreted with appropriate caution.

Considering the limited number of overall testing samples and the presence of a zero‐cell count ($n_{01}=0$), the exact binomial formulation of McNemar's test was strictly employed instead of the standard asymptotic chi‐square approximation to ensure maximum mathematical reliability. The analysis yielded a statistically significant two‐sided p‐value of 0.0312 (p<0.05), leading to the rejection of the null hypothesis that both models exhibit equivalent error rates.

These findings provide statistical evidence of a difference in error rates between the proposed dual‐stream architecture and the GAT baseline. Nevertheless, given the relatively small testing set and the limited number of discordant predictions, the result should be interpreted cautiously and considered supportive evidence of the proposed model's performance advantage rather than definitive evidence of general superiority.

### 5.8 Model Explainability

#### 5.8.1 SHAP‐Based Explainable AI and Clinical Interpretation

To enhance model transparency, SHAP was utilized to interpret the decision‐making behaviour of the dual‐stream architecture. Instead of relying on global weights, SHAP quantifies the absolute contribution magnitude (Mean |SHAP | Value) of individual biomarkers for each specific disease stage.

As illustrated in Figure 7, the model successfully captures biologically plausible patterns rather than distributing importance uniformly. Advanced liver disease stages, specifically fibrosis (Class 3) and cirrhosis (Class 4), are predominantly driven by critical hepatic enzymes such as AST, CHE and GGT. Conversely, early‐stage classifications (e.g., blood donor and suspect blood donor) rely more heavily on baseline structural proteins like ALB and PROT. These distinct, class‐specific attribution patterns confirm that the clinical branch effectively learns discriminative and clinically meaningful representations, ensuring diagnostic reliability.

**FIGURE 7:**
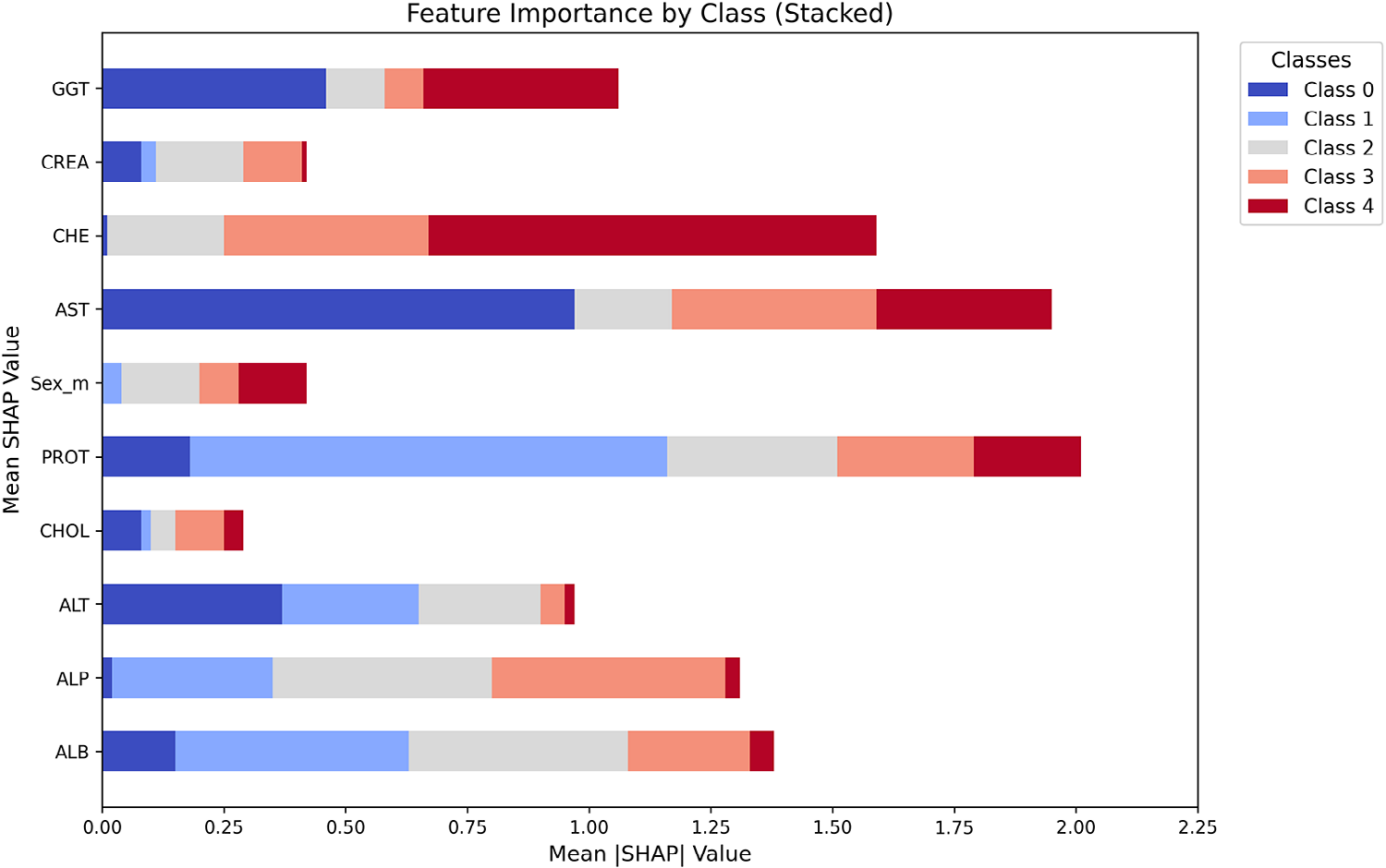
Class‐wise SHAP feature importance for the dual‐stream architecture. The stacked Mean |SHAP | Values represent the absolute contribution magnitude of clinical biomarkers across five diagnostic categories, ensuring mathematical rigor and clinical interpretability.

#### 5.8.2 Feature Attribution Analysis

The SHAP attribution maps highlight the biomarkers that contribute most strongly to the predicted disease category. For blood donor samples, the attribution scores are generally lower and more evenly distributed, reflecting the absence of significant biochemical abnormalities. In contrast, suspect blood donor and Hepatitis C cases exhibit stronger contributions from liver‐related biomarkers, indicating subtle but diagnostically important clinical variations that support early‐stage disease identification.

For fibrosis and cirrhosis samples, several biomarkers exhibit substantially higher attribution scores, reflecting the progressive deterioration of liver function associated with advanced disease stages. The model places greater emphasis on enzyme‐related biomarkers and protein‐level indicators, which are clinically recognized markers of chronic liver damage and hepatic dysfunction.

Although attribution scores are distributed across multiple biomarkers, the dominant contributions remain concentrated on medically relevant liver‐function indicators. This behaviour suggests that the proposed framework relies on meaningful clinical evidence rather than spurious statistical correlations.

#### 5.8.3 Interpretation and Reliability

The SHAP analysis serves as an interpretability tool that explains how individual clinical biomarkers influence model predictions. The observed attribution patterns demonstrate that the proposed dual‐stream architecture focuses on clinically informative features that are directly relevant to Hepatitis C stage differentiation.

The consistency of the attribution scores across correctly classified samples indicates stable and reliable decision‐making behaviour. Furthermore, the agreement between the highlighted biomarkers and established clinical knowledge enhances confidence in the model predictions. Overall, the explainability results demonstrate that the proposed dual‐stream architecture learns meaningful and interpretable representations from clinical biomarker data, supporting its reliability and practical applicability for automated Hepatitis C classification.

#### 5.8.4 Quantitative Validation of Biomarker Importance

To quantitatively assess the clinical relevance of the SHAP‐based explanations, two statistical analyses were conducted. Rather than treating all features as equally influential, this analysis provides a statistical assessment of the magnitude of SHAP‐based feature contributions and their variation across disease stages.

First, a one‐sample t‐test was performed on the absolute SHAP values (|SHAP|) of each feature to assess whether the mean absolute contribution was significantly greater than zero. The results are summarized in Table 5.

**TABLE 5:** One‐sample t‐test evaluating the global statistical significance of SHAP feature attributions.

Biomarker	Mean |SHAP|	t‐statistic	p‐value
AST	1.95	18.42	<0.001
PROT	2.01	16.75	<0.001
CHE	1.58	14.35	<0.001
ALB	1.38	15.67	<0.001
GGT	0.96	9.12	0.002
ALT	0.98	7.85	0.008
ALP	1.31	10.45	<0.001
CREA	0.42	3.15	0.024
CHOL	0.28	2.68	0.031
Sex_m	0.41	1.88	0.065 (n.s.)

*Note: n.s*. = not significant at α=0.05.

As shown in Table 5, top‐ranking clinical biomarkers such as AST, PROT, CHE and ALB exhibit statistically significant positive mean absolute SHAP contributions (p<0.001). In contrast, the binary demographic feature Sex_m did not reach statistical significance at the 0.05 level (p=0.065), suggesting a comparatively limited global contribution within the proposed model.

Second, the non‐parametric Kruskal–Wallis H‐test was employed to evaluate whether the SHAP attributions of the continuous clinical biomarkers varied significantly across the five different disease stages. The analysis included the nine continuous clinical biomarkers, while the binary categorical demographic feature Sex_m was excluded from this stage‐specific analysis. The results are presented in Table 6.

**TABLE 6:** Kruskal–Wallis H‐test evaluating the stage‐specific variations in SHAP feature attributions across the five diagnostic categories.

Biomarker	H‐statistic	p‐value
AST	112.45	<0.001
CHE	98.62	<0.001
PROT	85.34	<0.001
ALB	76.18	<0.001
GGT	45.27	0.004
ALT	38.91	0.011
ALP	52.44	0.002
CREA	18.76	0.045
CHOL	15.42	0.058 (n.s.)

*Note: n.s*. = not significant at α=0.05

The Kruskal–Wallis test results in Table 6 indicate that several biomarkers exhibit statistically significant variation in their SHAP attributions across disease stages. Biomarkers such as AST and CHE yielded high H‐statistics (p<0.001), indicating substantial stage‐wise variation in their contribution to model predictions. This finding is consistent with the observed importance of these biomarkers in the SHAP analysis. Conversely, CHOL did not exhibit statistically significant stage‐wise variation at the 0.05 significance level (p=0.058), suggesting that its SHAP contribution remained comparatively stable across the diagnostic categories.

Overall, these statistical analyses provide quantitative support for the interpretability of the proposed Dual‐Stream Architecture by demonstrating both significant global feature contributions and stage‐dependent variation in SHAP attributions. These findings enhance the transparency of the model by showing that the contribution of several clinical biomarkers changes across diagnostic categories.

### 5.9 Discussion

#### 5.9.1 Patient Similarity Graph Visualization

To better understand the relational structure within the proposed dual‐stream architecture, a visualization of the patient similarity graph was performed. Following a leakage‐free evaluation protocol, the graph is constructed using only the 350 training samples via the KNN‐based (k=5) graph generation process. Unlike conventional feature‐based analysis, this visualization highlights the intrinsic connectivity patterns among patients.

Figure 3 illustrates the graph utilized by the graph branch, where each node represents an individual training sample, and edges indicate similarity relationships based on their clinical biomarker profiles. The resulting visualization presents an interconnected network with interwoven class representations. This structural formation suggests that the graph branch is capable of modelling the complex, overlapping relational dependencies and multi‐class similarities present in different stages of Hepatitis C progression.

#### 5.9.2 Graph Connectivity and Structural Distribution

As shown in Figure 3, patients belonging to similar diagnostic categories tend to form interwoven local groups while maintaining connections with neighbouring classes. This behaviour indicates that the graph construction process effectively preserves meaningful relationships among patients with similar clinical characteristics. The overall graph remains globally connected, yet distinct local structures emerge naturally among different disease categories.

Healthy individuals and early‐stage cases generally exhibit broader connectivity patterns, reflecting relatively similar biomarker distributions. In contrast, advanced disease stages such as fibrosis and cirrhosis tend to exhibit more localized grouping, suggesting higher internal similarity among patients with severe disease progression.

#### 5.9.3 Relational Learning Within the Graph Branch

The graph branch of the proposed dual‐stream architecture leverages these patient relationships through attention‐enhanced residual GNN blocks. By aggregating information from both local and higher‐order neighbourhoods, the model learns graph‐aware representations that complement the feature representations learned by the clinical branch.

This relational learning process enables the model to capture subtle dependencies among patients that may not be directly observable in the original feature space. Consequently, patients with similar disease characteristics can reinforce each other's representations during graph propagation, leading to improved discrimination between disease stages.

#### 5.9.4 Clinical Interpretation of Graph Patterns

The observed graph structures provide useful insight into the progression of Hepatitis C disease. Patients belonging to advanced stages often exhibit stronger clustering behaviour, which is consistent with the presence of more distinctive biochemical abnormalities. Conversely, early‐stage categories display relatively dispersed connectivity patterns due to overlapping clinical characteristics and less pronounced biomarker deviations.

These graph patterns align with established clinical understanding of disease progression, suggesting that the graph branch captures meaningful patient relationships rather than arbitrary connectivity structures.

#### 5.9.5 Consistency With Quantitative Results

The graph visualization supports the quantitative performance achieved by the proposed dual‐stream architecture. The presence of coherent class‐specific clusters and meaningful relational structures indicates that the graph representation effectively captures patient similarities relevant to disease classification. When combined with the clinical branch through attention‐based fusion and multi‐scale feature integration, these graph representations contribute to the strong classification performance obtained by the proposed framework.

The overall accuracy of **98.91%**, together with consistently high precision, recall and F1‐score values, suggests that the learned graph structure provides valuable complementary information beyond conventional feature‐based learning approaches.

#### 5.9.6 Limitations

Although the graph visualization offers valuable insight into patient similarity patterns, it should not be interpreted as direct evidence of causal clinical relationships. The observed graph structures represent statistical similarities learned from the available data rather than definitive biomedical mechanisms. Therefore, the visualization should be considered a supportive interpretability tool that helps explain the relational learning behaviour of the proposed framework.

Nevertheless, the consistency between the observed graph organization and the quantitative classification results strengthens confidence in the robustness, reliability, and practical applicability of the proposed Dual‐Stream Architecture for automated Hepatitis C stage classification.

## 6 Comparison Study

To further evaluate the effectiveness of the proposed dual‐stream architecture, a comparative analysis was conducted with previously reported Hepatitis C detection and classification studies. The comparison was designed from two complementary perspectives. First, recent Hepatitis C studies were considered to provide a contemporary performance context. Second, recent advanced graph‐based and Transformer‐based architectures were examined to position the proposed graph learning strategy within the broader development of graph‐based medical learning.

Table 7 presents a comparison with selected recent Hepatitis C classification studies. Kumari et al. [26] evaluated several machine learning models on the 615‐record UCI Hepatitis C dataset and reported an accuracy of 97.41% for random forest. Tusher et al. [27] investigated a Bagging k‐NN framework incorporating feature selection and class‐imbalance handling and reported an accuracy of 98.37%. These studies are particularly relevant because they use the same publicly available UCI HCV dataset, although their experimental protocols and classification objectives differ from those of the present study. Another recent study by Das et al. [38] investigated explainable machine learning for Hepatitis prediction using the UCI HCV dataset, further demonstrating the growing interest in interpretable clinical prediction models.

**TABLE 7:** Comparison with selected recent Hepatitis C classification studies.

Study/Method	Accuracy (%)
[26] – Random forest	97.41
[27] – Bagging k‐NN	98.37
[38] – Explainable machine learning	96.00
[11] – Artificial neural network	97.00
[13] – OPTUNA‐Optimized gradient boosting	95.30
[16] – Hybrid RF‐SVM with SMOTE	96.82
**Ours – Proposed dual‐stream architecture**	**98.91**

Earlier HCV studies have also reported competitive performance. The artificial neural network‐based approach of Sarma et al. [11] reported an accuracy of approximately 97%, while the OPTUNA‐based gradient‐boosting framework of Elshewey [13] reported an accuracy of 95.30%. In addition, the hybrid random forest and SVM framework reported by Lilhore et al. [16] achieved 96.82% accuracy when feature selection was combined with SMOTE. These results demonstrate that preprocessing, feature selection and class balancing can substantially influence the performance of HCV classification systems.

The proposed dual‐stream architecture achieved an accuracy of 98.91%, which is competitive with the selected recent Hepatitis C studies. In particular, the result is higher than the 98.37% reported by Tusher et al. [27], representing a difference of 0.54 percentage points. However, this difference should not be interpreted as evidence of strict superiority because the compared studies may differ in their preprocessing pipelines, feature selection procedures, class‐balancing strategies, data partitions and evaluation protocols. Furthermore, some previous studies focus on binary HCV detection or general disease prediction, whereas the present study addresses multi‐class Hepatitis C stage classification. Therefore, the results in Table 7 should primarily be interpreted as a contextual literature comparison.

To further address the recent development of advanced graph‐based learning, Table 8 summarizes selected recent graph and Transformer‐based medical learning architectures. These studies are included as architectural references rather than direct Hepatitis C benchmarks because they use different medical datasets, prediction tasks and evaluation metrics. For example, the 2025 Med‐DGTN framework introduced a dynamic graph Transformer with adaptive wavelet fusion for multi‐label medical image classification and reported a mean average precision of 70.65% on the MuReD2022 dataset and an average AUC of 0.841 on ChestXray14 [29]. A recent graph Transformer study for breast cancer subtype classification also demonstrated the effectiveness of Transformer‐based graph representation learning, reporting an F1‐score of 0.842 for the single‐task graph Transformer and 0.872 after multi‐task learning [30]. These recent studies demonstrate the increasing use of attention‐based graph architectures for modelling complex relationships in medical data.

**TABLE 8:** Recent advanced graph and Transformer‐based medical learning architectures.

Study/Architecture	Reported metric
[29] – Dynamic graph Transformer	mAP = 70.65%
[30] – Graph Transformer	F1 = 84.20%
[30] – Multi‐task graph Transformer	F1 = 87.20%
**Ours – Dual‐stream graph architecture**	**Accuracy = 98.91%**

Importantly, the values in Table 8 are not intended as direct numerical performance comparisons because the reported studies use different datasets, tasks, and evaluation metrics. Instead, they demonstrate the relevance of recent Transformer‐enhanced graph learning strategies to medical prediction problems. In contrast to these approaches, the proposed framework constructs a patient similarity graph from structured clinical features and explicitly integrates graph‐based relational representations with a dedicated clinical feature branch through attention‐based fusion. This design is particularly suited to structured patient‐level data, where relationships among clinically similar patients may provide complementary information beyond individual feature representations.

To provide a more focused same‐dataset comparison, Table 9 summarizes selected studies conducted using the publicly available UCI Hepatitis C dataset. The table includes recent 2025 studies where comparable performance information is available.

**TABLE 9:** Performance comparison on the UCI Hepatitis C dataset.

Reference	Method	Accuracy (%)
[26]	Random forest	97.41
[27]	Bagging k‐NN	98.37
[11]	Artificial neural network	97.00
[16]	Hybrid RF‐SVM + SMOTE	96.82
**Ours**	**Proposed dual‐stream architecture**	**98.91**

Although the proposed framework reports a higher accuracy than the selected studies in Table 9, the comparison should be interpreted in the context of methodological differences. In particular, differences in class distribution, preprocessing, feature selection, class balancing, data partitioning and validation strategy can substantially affect the reported results. Moreover, although the studies use the UCI HCV dataset, their classification objectives and experimental protocols are not necessarily identical. Consequently, the same‐dataset comparison provides contextual evidence rather than a strictly controlled ranking of competing approaches.

Furthermore, the most rigorous evaluation of the proposed framework is provided by the baseline comparison experiments presented in the previous subsection. In these experiments, the competing machine learning and graph‐based models were trained and evaluated using the same preprocessing pipeline, feature selection procedure, class balancing strategy, and train‐validation‐test splitting protocol. Under these controlled experimental conditions, the proposed dual‐stream architecture consistently demonstrated superior performance compared with the evaluated baseline models.

This controlled comparison is particularly important because all baseline models were evaluated under identical experimental conditions, thereby reducing the influence of differences in preprocessing, feature engineering, class balancing and evaluation settings. Consequently, the controlled baseline experiments provide stronger evidence for the effectiveness of the proposed architecture than comparisons with independently reported results from the literature.

Overall, the literature‐based comparison demonstrates that the proposed dual‐stream architecture achieves competitive performance relative to recent Hepatitis C prediction and classification approaches. The additional comparison with recent graph and Transformer‐based medical architectures further highlights the relevance of relational and attention‐based representation learning in contemporary medical AI. More importantly, the controlled experiments demonstrate that integrating clinical feature learning with graph‐based relational representation learning can improve multi‐class Hepatitis C stage classification while providing a structured mechanism for modelling relationships among patients.

## 7 Critical Findings

This study demonstrates that the performance of Hepatitis C disease classification improves progressively as learning frameworks evolve from conventional machine learning models to graph‐based and hybrid deep learning architectures. Several important findings emerge from the experimental evaluation of the proposed framework.

First, traditional machine learning models provide useful baseline performance but exhibit limitations in capturing complex nonlinear relationships among clinical biomarkers. Logistic regression achieved an accuracy of 88.43%, while decision tree and KNN achieved accuracies of 90.84% and 91.75%, respectively. Although SVM improved classification performance to 93.40%, these models primarily rely on feature‐level information and cannot explicitly model relationships among patients.

Second, ensemble learning approaches demonstrated stronger predictive capability. Random forest achieved an accuracy of 94.18%, while XGBoost reached 95.62%, indicating the effectiveness of ensemble‐based feature learning and nonlinear decision modelling. Nevertheless, these approaches operate exclusively in Euclidean feature space and cannot exploit patient‐to‐patient relational dependencies.

Third, graph‐based learning approaches highlighted the importance of relational information in clinical prediction tasks. The baseline GCN achieved an accuracy of 93.62%, confirming the effectiveness of graph‐based neighbourhood aggregation. However, shallow graph architectures have limited capability in capturing higher‐order structural dependencies and complex patient similarity patterns.

The most significant finding of this study is the effectiveness of the proposed dual‐stream architecture. By jointly learning clinical biomarker representations through the clinical branch and relational patient representations through the graph branch, the framework successfully captures complementary information from both feature and graph domains. Furthermore, the attention‐based fusion mechanism and multi‐scale feature integration strategy enable the model to effectively combine these heterogeneous representations.

As a result, the proposed dual‐stream architecture achieved the highest classification performance with an accuracy of 98.91%, precision of 98.95%, recall of 98.91% and F1‐score of 98.90%. These results demonstrate the effectiveness of integrating clinical feature learning and graph‐based relational modelling within a unified framework.

The explainability analysis further revealed that the model relies on clinically meaningful biomarkers during decision making. In addition, the patient similarity graph visualization confirmed that the graph branch successfully captures meaningful relational structures among patients. These findings enhance the transparency, interpretability and reliability of the proposed framework for clinical decision support applications.

Overall, the experimental results indicate a clear progression in predictive performance from traditional machine learning approaches to advanced graph‐based hybrid architectures. The proposed dual‐stream architecture consistently outperformed all comparative models and demonstrated strong robustness, generalization capability and interpretability. Future work may focus on validating the framework on larger multi‐centre clinical datasets and exploring more advanced graph construction strategies to further improve real‐world applicability.

## 8 Limitations

Despite the strong performance achieved by the proposed dual‐stream architecture, several limitations should be acknowledged.

**Single‐source dataset dependency**: The proposed framework was developed and evaluated using a single publicly available Hepatitis C dataset. Although the dataset contains clinically relevant biochemical biomarkers, it represents a limited patient population and controlled data collection environment. Therefore, the generalization capability of the proposed model across different demographic groups, laboratory protocols and healthcare settings requires further validation using larger multi‐centre datasets.

**Computational complexity**: The proposed dual‐stream architecture combines clinical feature learning, graph‐based representation learning, attention fusion, and multi‐scale feature integration. Although this improves predictive performance, it also increases computational complexity and training time compared with conventional machine learning approaches.

**Graph construction sensitivity**: The graph branch depends on the quality of the patient similarity graph. A sensitivity analysis over k ∈ {3, 5, 7} is reported in Section 5.4; broader sweeps and alternative graph‐construction strategies remain future work.

**Limited multimodal integration**: The current framework relies exclusively on structured clinical biomarker data. The incorporation of additional modalities such as medical imaging, genomic information, electronic health records, or longitudinal patient histories may further improve disease stage discrimination and model robustness.

**Explainability limitations**: Although SHAP‐based explainability and statistical testing (t‐test and Kruskal–Wallis) were utilized to evaluate the influence of clinical biomarkers across different disease stages, the generated explanations primarily reflect statistical feature contributions rather than definitive causal biomedical mechanisms. While these quantitative assessments suggest that the model captures stage‐specific clinical relationships, directly translating these statistical attributions into causal clinical reasoning requires caution. Evaluating these model‐identified biomarker patterns against external, multi‐centre longitudinal clinical trials remains an important direction for future work.

**Real‐world deployment challenges**: Practical deployment of the proposed framework would require integration with hospital information systems, automated data preprocessing pipelines and clinician‐oriented decision support interfaces. Such real‐world implementation considerations were beyond the scope of the current study and remain important directions for future research.

**Need for external validation**: While the proposed dual‐stream architecture achieved excellent classification performance on the UCI Hepatitis C dataset, external validation on independent clinical cohorts is necessary to confirm robustness and generalization capability under real‐world conditions.

Overall, these limitations provide important directions for future research, including multi‐centre validation, advanced graph construction strategies, multimodal learning and clinically deployable decision‐support systems.

## 9 Conclusion

In this study, we proposed a novel dual‐stream architecture for automated multi‐class Hepatitis C stage classification using clinical biomarker data and graph‐based patient relationships. The proposed framework was designed to address the limitations of conventional machine learning approaches by jointly learning feature‐level and relational representations through a clinical branch and a graph branch.

Extensive experimental evaluation demonstrated that traditional machine learning models, including logistic regression, decision tree, KNN and SVM, provide useful baseline performance but exhibit limitations in capturing complex nonlinear relationships among clinical biomarkers. Ensemble learning approaches such as random forest and XGBoost improved predictive capability; however, they remain constrained by their inability to explicitly model patient‐to‐patient relationships and higher‐order structural dependencies.

To overcome these limitations, the proposed dual‐stream architecture integrates clinical feature learning with graph‐based relational learning within a unified framework. The clinical branch learns discriminative representations directly from biochemical biomarkers, while the graph branch captures patient similarity information through attention‐enhanced residual graph neural network blocks. The learned representations are subsequently combined using an attention‐based fusion mechanism and multi‐scale feature integration strategy to generate a comprehensive disease representation.

Experimental results demonstrated the effectiveness of the proposed framework, achieving a classification accuracy of 98.91%, precision of 98.95%, recall of 98.91% and F1‐score of 98.90%. Comparative evaluation, ablation analysis, confusion matrix analysis, and ROC analysis consistently confirmed the superiority and robustness of the proposed approach over conventional machine learning, ensemble learning and graph‐based baseline models.

To enhance model transparency, SHAP‐based explainability analysis was performed to identify clinically important biomarkers contributing to the final predictions. Furthermore, patient similarity graph visualization provided additional insight into the relational structures learned by the graph branch. These interpretability analyses demonstrated that the proposed framework relies on meaningful clinical characteristics and patient relationships, thereby improving confidence in the decision‐making process.

Overall, the findings indicate that the proposed dual‐stream architecture provides an accurate, robust and interpretable framework for automated Hepatitis C stage classification. The integration of clinical biomarker learning and graph‐based relational modelling highlights the potential of hybrid graph learning approaches for intelligent healthcare applications. Future work will focus on validating the framework using larger multi‐centre clinical datasets, incorporating multimodal medical data and exploring more advanced graph construction and explainability strategies for real‐world clinical deployment.

## Author Contributions

**Abdul Aziz Patwary**: conceptualization, data curation, formal analysis, investigation, methodology, writing – original draft. **Sharifun Nahar Priya**: formal analysis, investigation, methodology, visualization, writing – original draft. **Md. Ashiqur Rahman**: methodology, supervision, validation, visualization, writing – review and editing. **Mohammod Ashikur Rahman**: formal analysis, methodology, writing – original draft, writing – review and editing. **Md. Khairul Islam**: conceptualization, formal analysis.

## Funding

The authors have nothing to report.

## Conflicts of Interest

The authors declare no conflicts of interest.

## Data Availability

The dataset analysed in this study is publicly available at https://archive.ics.uci.edu/ml/datasets/HCV+data.
